# Fructose-Based Production of Short-Chain-Length and Medium-Chain-Length Polyhydroxyalkanoate Copolymer by Arctic *Pseudomonas* sp. B14-6

**DOI:** 10.3390/polym13091398

**Published:** 2021-04-26

**Authors:** Tae-Rim Choi, Ye-Lim Park, Hun-Suk Song, Sun Mi Lee, Sol Lee Park, Hye Soo Lee, Hyun-Joong Kim, Shashi Kant Bhatia, Ranjit Gurav, Kwon-Young Choi, Yoo Kyung Lee, Yung-Hun Yang

**Affiliations:** 1 Department of Biological Engineering, College of Engineering, Konkuk University, Hwayang-dong, Gwangjin-gu, Seoul 05029, Korea; srim1004@gmail.com (T.-R.C.); pyl6839@naver.com (Y.-L.P.); shs9736@naver.com (H.-S.S.); dltjsal6845@naver.com (S.M.L.); shckd2020@naver.com (S.L.P.); lhs2265696@naver.com (H.S.L.); sopero2@naver.com (H.-J.K.); shashibiotechhpu@gmail.com (S.K.B.); rnjtgurav@gmail.com (R.G.); 2 Institute for Ubiquitous Information Technology and Applications, Konkuk University, Seoul 05029, Korea; 3 Department of Environmental and Safety Engineering, College of Engineering, Ajou University, Suwon-si 16499, Korea; kychoi@ajou.ac.kr; 4 Polar Research Institute, Incheon 21990, Korea; yklee@kopri.re.kr

**Keywords:** *Pseudomonas* strain, polyhydroxyalkanoate, fructose syrup, fermentation

## Abstract

Arctic bacteria employ various mechanisms to survive harsh conditions, one of which is to accumulate carbon and energy inside the cell in the form of polyhydroxyalkanoate (PHA). Whole-genome sequencing of a new Arctic soil bacterium *Pseudomonas* sp. B14-6 revealed two PHA-production-related gene clusters containing four PHA synthase genes (*phaC*). *Pseudomonas* sp. B14-6 produced poly(6% 3-hydroxybutyrate-*co*-94% 3-hydroxyalkanoate) from various carbon sources, containing short-chain-length PHA (scl-PHA) and medium-chain-length PHA (mcl-PHA) composed of various monomers analyzed by GC-MS, such as 3-hydroxybutyrate, 3-hydroxyhexanoate, 3-hydroxyoctanoate, 3-hydroxydecanoate, 3-hydroxydodecenoic acid, 3-hydroxydodecanoic acid, and 3-hydroxytetradecanoic acid. By optimizing the PHA production media, we achieved 34.6% PHA content using 5% fructose, and 23.7% PHA content using 5% fructose syrup. Differential scanning calorimetry of the scl-*co*-mcl PHA determined a glass transition temperature (T_g_) of 15.3 °C, melting temperature of 112.8 °C, crystallization temperature of 86.8 °C, and 3.82% crystallinity. In addition, gel permeation chromatography revealed a number average molecular weight of 3.6 × 10^4^, weight average molecular weight of 9.1 × 10^4^, and polydispersity index value of 2.5. Overall, the novel *Pseudomonas* sp. B14-6 produced a polymer with high medium-chain-length content, low T_g_, and low crystallinity, indicating its potential use in medical applications.

## 1. Introduction

Some microbes have developed various tactics to survive the harsh Arctic environment [1,2,3]. One of these survival mechanisms is to adapt to temperature and nutrition fluctuations by accumulating energy and carbon sources within the cell as polyhydroxyalkanoate (PHA) granules [4,5], which also function as chaperone-like molecules to protect internal cellular systems [6,7,8]. Although several microbes thrive at low temperatures and accumulate PHAs, the metabolism of Arctic bacteria is generally quite slow, weakening their potential as PHA producers [7].

PHAs are produced by bacteria using various renewable feedstocks, and are easily degraded under biological conditions [9]. Properties such as the monomer composition, distribution, and molecular weight of these biocompatible polyesters are controlled by different substrates or production hosts [10,11,12]. Depending on the desired property, production can be manipulated to yield different short-chain-length monomers such as butyrate and valerate, and medium-chain-length monomers such as hexanoate, octanoate, and chains of up to 14 carbon atoms in length [13,14,15,16]. Short-chain-length PHAs (scl-PHAs) such as polyhydroxybutyrate (PHB) have high rigidity but are brittle, while medium-chain-length PHAs (mcl-PHAs) are flexible and create a sticky surface, which provides them with thermoelastomeric properties suitable for biomedical applications such as in skin adhesives and drug delivery systems [13,17,18]. However, the production of mcl-PHAs has been relatively challenging due to difficulties in controlling the metabolic flux of the desired monomers [11,19].

*Pseudomonas* spp. are known for their ability to survive in wide-ranging temperatures and habitats, suggesting industrial potential thanks to their hydrolase activity and capacity for PHA and exopolysaccharide production [20]. *Pseudomonas* have been investigated as major PHA producers, usually producing mcl-PHA and rarely producing scl-PHA [21,22]. As the PHA polymer structure is affected by the substrates utilized during cultivation, the selection of the carbon source is important [23]. PHA production by *Pseudomonas* spp. using carbohydrate as a substrate usually leads to a low mcl-PHA yield, with a heterogeneous monomeric composition [24]. Selection of the proper substrate and optimization are needed in order to increase mcl-PHA yield and variety.

Although *Pseudomonas putida* is the most studied PHA-producing strain, species including *P. putida* and its variants have difficulty using sucrose directly [25]. Because sugarcane-based feedstocks and waste fructose syrup are cheap and abundant sources for PHA production, several efforts have been undertaken to metabolically engineer strains to fully uptake sucrose, pretreat feedstock to degrade sucrose to glucose and fructose, or discover strains that are able to utilize sucrose to produce PHA [25,26,27].

In the current study, we attempted to produce a unique PHA with a heterogenous monomer composition from the novel Arctic microbe *Pseudomonas* sp. B14-6. Media composition and culture conditions such as temperature, inoculum size, and cultivation time were optimized to achieve high PHA production and content. Additionally, we cultured *Pseudomonas* sp. B14-6 under optimal conditions with fructose, a single monosaccharide, and fructose syrup, a cost-effective complex carbon source. Finally, we analyzed the physical properties of the produced PHA to hypothesize possible applications of the strain and the PHA.

## 2. Materials and Methods

### 2.1. Chemicals

All chemicals used in the present study were suitable for microbial culture or of analytical grade. Glucose, fructose, sucrose, galactose, lactose, glycerol, N-acetylglucosamine, and xylose were purchased from Sigma-Aldrich (St. Louis, MO, USA). Other chemicals used in the growth media were also purchased from Sigma-Aldrich or BD Difco (Franklin Lakes, NJ, USA). GC-MS authentic 3-hydroxybutyrate (3HB), 3-hydroxyhexanoate (3HHx), 3-hydroxyoctanoate (3HO), 3-hydroxydecanoate (3HD), 3-hydroxydodecanoate (3HdD), and 3-hydroxytetradecanoate (3HtD) were also purchased from Sigma-Aldrich.

### 2.2. Whole-Genome Sequencing and BLAST Search for PHA Genes

The newly isolated Arctic soil bacterium *Pseudomonas* sp. B14-6 was whole-genome sequenced by Cosmogenetech (Seoul, Korea), and the sequencing data was deposited in Genbank for assignment (accession number CP053929), and was also deposited in BioProject (ID PRJNA634457) [28]. To determine which genes were involved in PHA production, a BLAST search was conducted using reference genes and PHA-synthesis-related gene clusters.

### 2.3. Strain and Culture Conditions

The seed culture of *Pseudomonas* sp. B14-6 was cultured in Luria-Bertani broth (LB) at 200 rpm and 30 °C. The standard PHA production media consisted of 6.78 g/L Na_2_HPO_4_, 3 g/L KH_2_PO_4_, 0.5 g/L NaCl, 1 g/L NH_4_Cl, 20 g/L carbon source, and 1 g/L yeast extract. The inoculum size was 1% of the final volume, cultivated at 30 °C and 200 rpm shaking [29]. The optimization procedure altered the media composition (carbon source, nitrogen source, and their concentrations), inoculum size, cultivation time, and incubation temperature. Flask experiments used 50 mL working volume in 250 mL total volume baffled flask and orbital shaking at 200 rpm.

### 2.4. PHA Analysis

The PHA composition of *Pseudomonas* sp. B14-6 was evaluated using a previously described GC-MS method [30]. Strains were cultured in PHA production media, the culture was centrifuged (3500× *g* for up to 30 min at 4 °C), and the cells were washed twice with deionized water. The washed cells were transferred to a glass vial for lyophilization. Fatty acid methyl ester (FAME) derivatization was conducted to prepare the sample for GC-MS, as previously described [31,32]. Briefly, 1 mL of 15% sulfuric-methanol and 1 mL of chloroform were added to the lyophilized sample, followed by heating for 2 h at 100 °C. The sample was then held at room temperature for 15 min and 1 mL of distilled water was added. The sample was vortexed, the chloroform layer was transferred to a new tube containing NaSO_4_, and the sample was filtered into a clean borosilicate glass tube. The resulting sample was analyzed by GC-MS (Perkin Elmer, Waltham, MA, USA) equipped with a fused silica capillary column (Elite-5 ms (Perkin Elmer); 30 m × 0.25 mm i.d. × 0.25 μm) and subjected to a linear temperature gradient for PHA (50 °C for 1 min, increased at 15 °C/min to 120 °C for 2 min, and then increased at 10 °C/min to 300 °C for 10 min). The injector port temperature was set at 250 °C. Mass spectra were obtained by electron impact ionization at 70 eV, and scan spectra were obtained within the range of 45–450 m/z. Selected ion monitoring was used for the detection and fragmentation analysis of the major products [33]. All PHA authentic samples from 3HB to 3HtD were derivatized as methyl esters and applied to GC-MS to confirm the retention time and mass spectrum of each compound.

### 2.5. Physical Properties of Produced PHA

Analysis of PHA produced by *Pseudomonas* sp. B14-6 was conducted using several methods. The thermal properties of PHA were investigated by differential scanning calorimetry (DSC; TA Instruments, New Castle, DE, USA), using temperatures ranging from −60 °C to 180 °C, increasing at 10 °C/min. Gel permeation chromatography (GPC; Young Lin Instrument Co., Anyang, Korea) equipped with a K-804 column was used to measure the molecular weight of the copolymer. A PHA sample (0.1 mg) was dissolved in 1 mL of chloroform and injected in the GPC instrument at 40 °C oven temperature for 30 min. Results were analyzed based on a calibration curve using Shodex polystyrene standards SM-105 (Tokyo, Japan), which have a molecular weight range from 6530 Da to 2330 kDa [34].

## 3. Results and Discussion

### 3.1. Screening of PHA-Related Genes in Pseudomonas sp. B14-6

**Figure 1:**
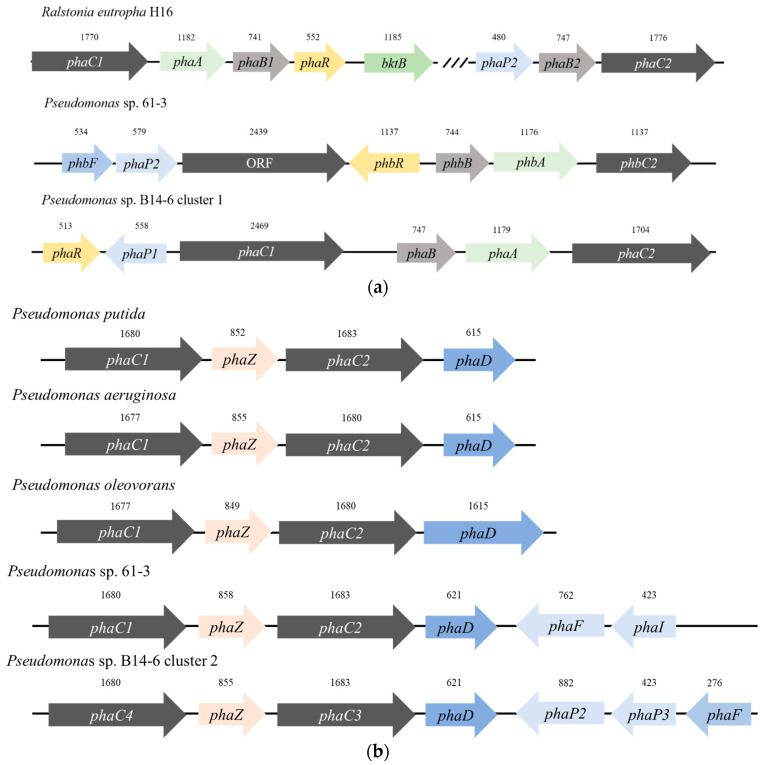
PHA production gene clusters of *Pseudomonas* sp. B14-6. (**a**) Comparison of PHA synthesis cluster of *Ralstonia eutropha* H16, *Pseudomonas* sp. 61-3, and first PHA synthesis cluster of *Pseudomonas* sp. B14-6. (**b**) Comparison of PHA synthesis cluster of PHA-producing *Pseudomonas* species that have type-II PHA synthase to the second PHA synthesis cluster of *Pseudomonas sp.* B14-6. *phaA* encodes β-ketothiolase; *phaB* encodes acetoacetyl-CoA reductase; *phaC* encodes PHA synthase; *phaZ* encodes PHA depolymerase; and *phaD* and *phaR* control the simultaneous expression of PHA genes as transcriptional regulators.

We previously reported that whole-genome sequencing of *Pseudomonas* sp. B14-6 revealed a genome 6,776,772 base pairs in length. The strain showed dramatic membrane changes with temperature, and important players of membrane modification were identified [28]. Using the genome sequencing results, we identified two PHA-synthesis-related gene clusters and compared them with PHA-related genes from *Ralstonia eutropha*, *Pseudomonas,* and other PHA-producing species by BLAST (Figure 1). The two gene clusters had the orders *phaR-phaP1-phaC1-araC-phaB-phaA-phaC2* and *phaC4-phaZ-phaC3-phaD-phaP2-phaP3*, which shared high similarity with *Pseudomonas* sp. 61-3, although the annotation might differ [35]. Amongst these two PHA synthesis-related gene clusters, we identified four PHA synthase genes, named *phaC1*–*C4*, from the open reading frame order assigned by whole-genome sequencing. As PHA synthases can be classified as types I to IV [36], we attempted to classify each *phaC* by the BLAST search results.

One cluster had acetyl-CoA thiolase (*phaA)*, acetoacetyl-CoA reductase (*phaB*), and two PHB synthases (*phaC1* and *phacC2*), sharing high similarity with the PHA synthesis cluster of *Ralstonia eutropha* (a PHA synthase type-I that produces scl-PHA) (Figure 1a). The order of the PHA synthesis gene in *R. eutropha* is *phaC1-phaA-phaB1*; however, the order in *Pseudomonas* sp. B14-6 was *phaB-phaA-phaC2*, in the reverse order. We found that *phaC1* was a PHA synthetase, not a PHA synthase, with a much longer sequence than other *phaC* genes; it contained a PHA synthase type-IV-related domain, although its function was unknown. Next to *phaC1* was the *phaR* gene, known for the subunit required to produce PHA with *phaC* in the PHA synthase type-IV system. However, it was difficult to determine its specific function because *phaC* had a longer sequence than the PHA synthase type-IV of *Bacillus megaterium,* and was similar to that of *Pseudomonas* sp. 61-3 [36]. In addition, the two *PhaC* genes in *R. eutropha* are located very far apart, with one cluster having the order *phaC1-phaA-phaB1-phaR-bktB* (β-ketothiolase) and the other having the order *phasin2-phaC2-phaB2*. The gene cluster of *Pseudomonas* sp. B14-6 was thus unique, with both *phaC* genes at neighbor locations.

The other PHA gene cluster also had two *phaC* genes (*phaC3* and *phaC4)* and a PHA depolymerase (*phaZ*) resembling a PHA synthase type-II that appears in typical PHA-producing *Pseudomonas* spp. Compared with the PHA gene cluster of *Pseudomonas* spp., the length of the *phaC3* gene was the same as that of *Pseudomonas putida*, and the *phaD* was slightly longer than that of *P. putida* and *Pseudomonas aeruginosa*, but shorter than the long *phaD* gene of *Pseudomonas oleovorans* (Figure 1b). As both *Pseudomonas* sp. 61-3 and *Pseudomonas* sp. B14-6 have similar gene clusters and two different types of PHA synthesis gene clusters for scl-PHAs (*phaC2)* and mcl-PHAs (*phaC3* and *phaC4*), it was expected that *Pseudomonas* sp. B14-6 would produce similar PHAs as *Pseudomonas* sp. 61-3 or *Pseudomonas* sp. MPC6.

### 3.2. Analysis of PHA from Pseudomonas sp. B14-6

According to the gene search results, we focused on two points: (1) *Pseudomonas* sp. B14-6 could produce PHA, and (2) PHA produced by the strain might contain monomeric units, both medium and short chain length.

**Figure 2:**
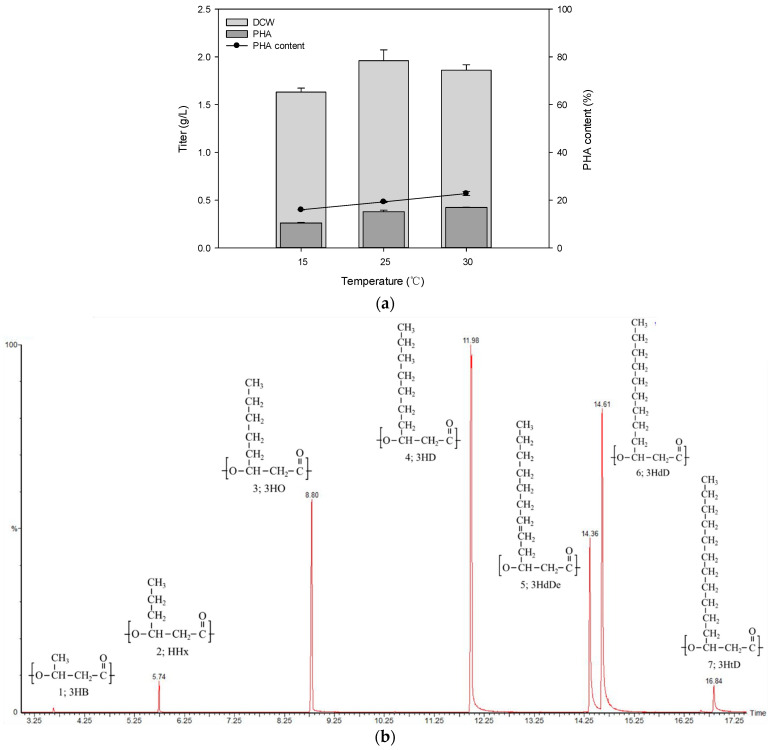
PHA production of *Pseudomonas* sp. B14-6 at different temperatures, and monomer composition after cultivation for 120 h. (**a**) DCW, PHA titer, and PHA content of *Pseudomonas* sp. B14-6 at different temperatures. (**b**) *Pseudomonas* sp. B14-6-produced PHA monomer composition chromatogram analyzed by GC-MS. Peak assignments: 1: 3HB, 2: 3HHx, 3: 3HO, 4: 3HD, 5: 3HdDe, 6: 3HdD, and 7: 3HtD. DCW, dry cell weight.

As the growth temperature range of *Pseudomonas* sp. B14-6 is between 4 °C and 30 °C, we first measured growth and PHA accumulation at different temperatures in standard PHA production media. Several studies have reported that low temperatures could stimulate higher PHA content in single cells, but relatively low biomass. However, when *Pseudomonas* sp. B14-6 was cultured at 15 °C, 25 °C, and 30 °C, dry cell weight (DCW) was highest at 25 °C and PHA content was highest at 30 °C (Figure 2a). Although *Pseudomonas* sp. B14-6 was isolated from Arctic soil and could grow at low temperatures, a moderate temperature of 30 °C was optimal for PHA production. The produced PHA was analyzed by GC-MS, revealing seven different monomeric units containing 3HB as a short-chain monomeric unit; 3HHx, 3HO, 3HD, 3HdD, and 3HtD as medium-chain monomeric units; and 3-hydroxy-5-*cis*-dodecenoic acid (HdDe) as an unsaturated monomeric unit (Figure 2b). The ratio of monomers in poly(6.0% 3HB-*co*-8.5% 3HHx-*co*-39.8% 3HO-*co*-33.6% 3HD-*co*-5.6% 3HdDe-*co*-6.5% 3HdD-*co*-0.1% 3HtD) differed from that in *Pseudomonas* sp. 61-3 poly(44% 3HB-*co*-5% 3HHx-*co*-21% 3HO-*co*-25% 3HD-*co*-3% 3HdD-*co*-3% 3HdDe) and *Pseudomonas* sp. MPC6 poly(89.5% 3HB-*co*-1.8% 3HHx-*co*-3.3% 3HO-*co*-4.4% 3HD-*co*-1.1% 3HdD) [37,38]. *Pseudomonas* sp. B14-6 produced a very high proportion of medium-chain-length monomers (96% of PHA content), compared with 56% and 89.5% from *Pseudomonas* sp. 61-3 and *Pseudomonas* sp. MPC6, respectively [39,40]. It was expected that the physical properties of the PHA produced by *Pseudomonas* sp. B14-6 would differ from those of previously reported PHAs.

### 3.3. Optimization of mcl-PHA Production

**Figure 3:**
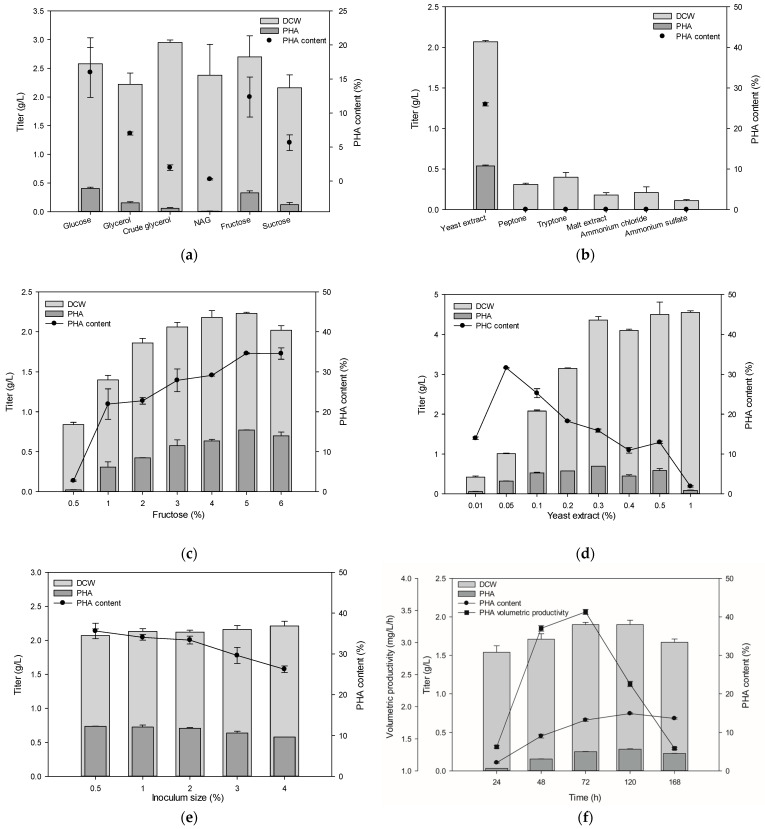
Optimization of media. (**a**) Carbon sources including glucose, fructose, sucrose, glycerol, crude glycerol, and N-acetyl glucosamine. (**b**) Nitrogen sources including yeast extract, malt extract, ammonium chloride, ammonium sulfate, peptone, and tryptone (0.1%). (**c**) Fructose concentration from 1% to 6%. (**d**) Yeast extract concentration from 0.05% to 1%. (**e**) Inoculum size from 0.5% to 4%. (**f**) Cultivation time from 24 to 168 h. DCW, dry cell weight.

To determine the optimal nutrients for mcl-PHA production, we screened various carbon sources, including glucose, fructose, xylose, N-acetyl glucosamine, galactose, lactose, and sucrose, and compared monomeric composition from the carbon source (Table 1). Among them, we focused on fructose as the main carbon source (Figure 3a). Additionally, we screened yeast extract, malt extract, peptone, and tryptone as complex nitrogen sources, as well as ammonium sulfate and ammonium chloride as defined nitrogen sources. Among these, yeast extract yielded the highest PHA content (Figure 3b). Further optimization was conducted using fructose as the carbon source and yeast extract as the nitrogen source. We determined that 5% fructose yielded the highest PHA content of 36% (Figure 3c), while optimal biomass and PHA content were achieved with 0.1% yeast extract (Figure 3d). Another parameter that affects PHA production is the amount of microbes inoculated in the media. Thus, we evaluated PHA production using inoculum at 0.5% to 4% of the final volume, determining 0.5% as the optimal inoculum size (Figure 3e). PHA production is also affected by cultivation time, in which gene expression and polymer building is not completed during a short cultivation, and accumulated PHA is degraded as an energy source due to exhaustion of the initial carbon source during a long cultivation. Therefore, cultivation time for PHA production was evaluated for 168 h, revealing that culture for 120 h yielded optimal biomass and PHA content, but volumetric productivity was the highest at 48 h (Figure 3f). The composition of PHA was changed by different temperatures (data not shown). As a result, the optimal conditions for PHA production with *Pseudomonas* sp. B14-6 were set as 5% fructose, 0.1% yeast extract, 0.5% inoculum size, and 120 h culture time at 30 °C. PHA production in optimal conditions was higher than other psychrophilic *Pseudomonas* spp. (Table 2).

### 3.4. Monitoring Time-Dependent PHA Production and Fructose Syrup Application

**Figure 4:**
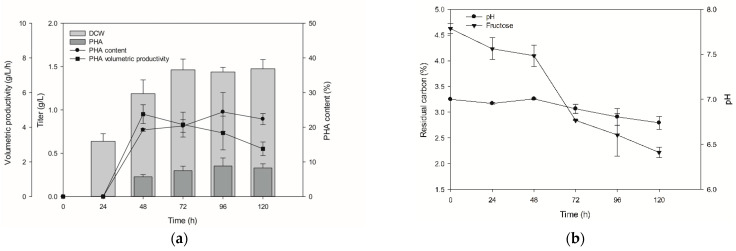

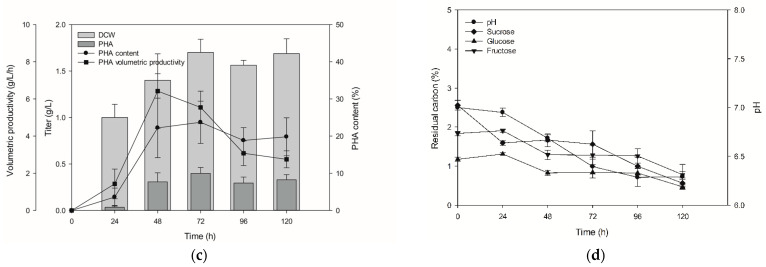
**Monitoring of PHA production at different time points under the application of fructose and fructose syrup.** (**a**) Time-dependent PHA production of *Pseudomonas* sp. B14-6 with optimized conditions. (**b**) Measurement of residual fructose and pH. (**c**) Time-dependent PHA production supplemented with fructose syrup as a carbon source. (**d**) Time-dependent residual sugars from fructose syrup and pH change. DCW, dry cell weight.

The time-dependent production of PHA by *Pseudomonas* sp. B14-6 was investigated under optimized conditions, measuring DCW, amount of PHA produced, and carbon source consumption over 120 h. Almost no PHA was produced during the first 24 h, but both PHA content and DCW increased from 48 h until 72 h. DCWs at 72 h and 120 h were similar and volumetric productivities were slightly decreased, but the highest PHA content was obtained at 96 h (Figure 4a). The consumption of fructose increased after 48 h, and the pH began decreasing from approximately 7 to 6.69 at 120 h (Figure 4b). We then evaluated the use of fructose syrup composed of 20% glucose, 36% fructose, and 44% sucrose as an economical carbon source. The production of PHA with media that contained 5% fructose syrup achieved 23.7% PHA content (Figure 4c). Use of fructose syrup induced a different pH pattern, optimal culture time, and lower PHA content than using fructose (Figure 4d). No catabolite repression occurred during cultivation, as all sugars were consumed together.

### 3.5. Physical Properties of Produced PHA

**Figure 5:**
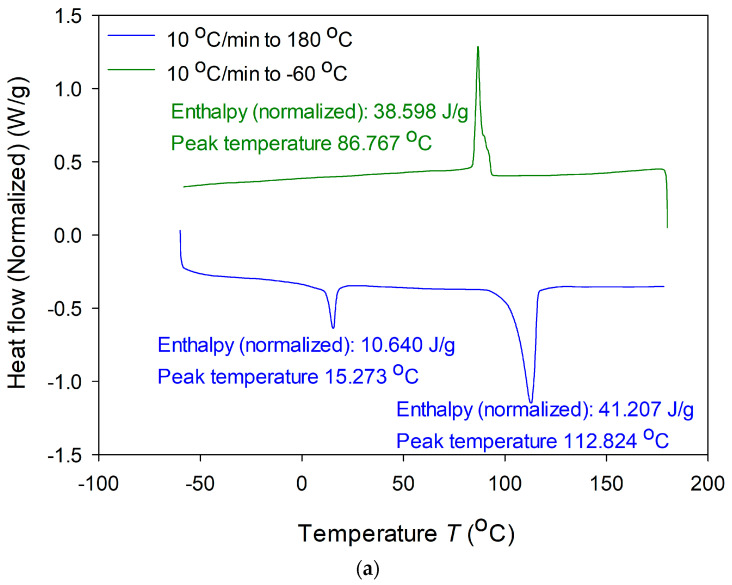

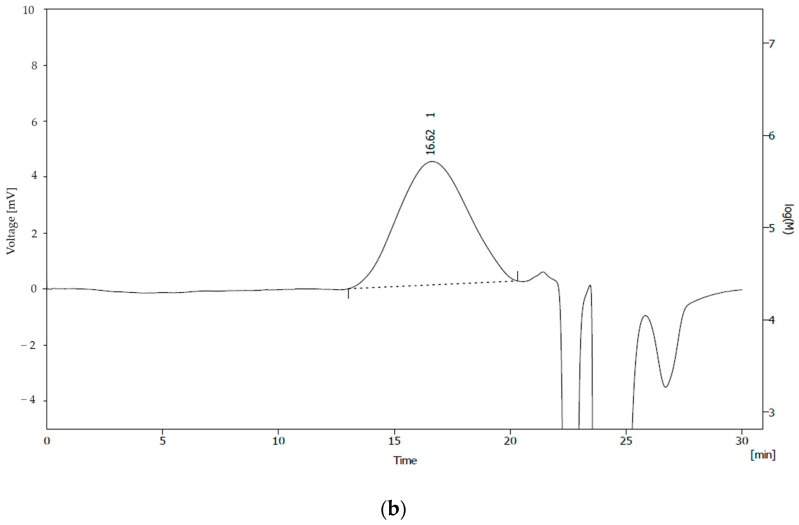
Physical analysis of scl-*co*-mcl PHA produced by *Pseudomonas sp.* B14-6 by (**a**) differential scanning calorimetry and (**b**) gel permeation chromatography.

The thermal properties of poly(5.0% 3HB-*co*-7.8% 3HHx-*co*-25.6% 3HO-*co*-40.0% 3HD-*co*-9.9% 3HdDe-*co*-11.0% 3HdD-*co*-0.7% 3HtD) were investigated by DSC, including the glass transition temperature (T_g_), melting temperature (T_m_), crystallization temperature (T_c_), and melting enthalpy (ΔH_m_). For the scl-*co*-mcl PHA, T_g_ = 15.3 °C, T_c_ = 86.8 °C with an enthalpy = 38.6 J/g, and T_m_ = 112.8 °C with ΔH_m_ = 41.2 J/g were obtained (Figure 5a).

The thermal characteristics of our scl-*co*-mcl PHA differed both in T_m_ and T_c_ from those of the previously reported mcl-PHA from *Pseudomonas* sp. PAMC28620 composed of poly(25.5% 3HO-*co*-52.1% 3HD-*co*- 5.7% 3HdD-*co*-16.7% 3HtD) with T_m_ = 172.8 °C, T_g_ = 3.99 °C, and T_c_ = 54.61 °C [30]. The thermal properties also differed from those of the mcl-PHA from *Pseudomonas* sp. MPC6 composed of poly(89.5% 3HB-*co*-1.8% 3HHx-*co*-3.3% 3HO-*co*-4.4% 3HD-*co*-1.1% 3HdD) with T_m_ = 163.5 °C, T_g_ = 2.3 °C, and T_c_ = 46.0 °C [37]. The scl-*co*-mcl PHA from *Pseudomonas* sp. B14-6 had lower T_m_ values and higher T_g_ and T_c_ values due to the low 3HB content and high 3HO and 3HD content, which lowered the melting point. As an important polymer parameter, the degree of crystallinity (X_c_) was calculated from the enthalpy, revealing 3.82% crystallinity. This X_c_ value was extremely low compared to that reported for mcl-PHAs from *Pseudomonas* sp. PAMC28620 (X_c_ = 43.7%) [30], *Bacillus thermoamylovorans* PHA005 (X_c_ = 43.0%) [44], and *R. eutropha* (X_c_ = 91%) [45]. As highly crystalline polymers have limited application in industrial and medical fields [46], the scl-*co*-mcl PHA from *Pseudomonas* sp. B14-6 may have potential in applications that require a sticky and relatively low-temperature modeling polymer, as well as biodegradable characteristics.

The molecular weight of the obtained PHA was characterized by GPC, demonstrating a retention time peak start at 13.02 min, peak maximum at 16.62 min, and peak end at 20.32 min (Figure 5b). The scl-*co*-mcl PHA had average values, with a number average molecular weight (M_n_) of 3.6 × 10^4^, weight average molecular weight (M_w_) of 9.1 × 10^4^, Z-average (M_z_) of 1.9 × 10^5^, and viscosity average molar mass (M_v_) of 8.0 × 10^4^. The polydispersity index value of scl-*co*-mcl PHA was 2.5, which differed from other values of scl-PHA or mcl-PHA, due to the mixed monomeric unit composition (Table 3). The high polydispersity index value was due to the various monomer unit composition and breakdown of the polymer in the sample preparation steps. The physical properties of the scl-*co*-mcl PHA produced by *Pseudomonas* sp. B14-6 differed from PHAs produced by other species. Therefore, we hypothesize that the scl-*co*-mcl PHA has potential applications in biomedical or similar industrial fields.

## 4. Conclusions

PHA has attracted attention as an alternative material to replace petroleum plastics. The composition of monomers, especially medium-chain-length monomers such as 3HO and 3HD, influences the properties of the polymers, decreasing the rigidity and high crystallinity of PHB and increasing the toughness of the polymer. *Pseudomonas* species have been studied as possible mcl-PHA producers, including many mesophile *Pseudomonas* strains. We previously isolated the novel Arctic strain *Pseudomonas* sp. B14-6, which shared a similar gene structure with *Pseudomonas* sp. 61-3 and contained two different gene clusters for scl-PHB and mcl-PHA. Although the two species had similar gene clusters and alignments, the new strain produced a PHA with a different monomer ratio than previously reported PHAs. In particular, the strain produced 94% medium-chain-length monomer content, compared with less than 60% for *Pseudomonas* sp. 61-3. As a result, the scl-*co*-mcl PHA from *Pseudomonas* sp. B14-6 exhibited totally different T_m_, T_g_, and T_c_ values from other reported PHAs. Furthermore, *Pseudomonas* sp. B14-6 was able to utilize a wide range of carbon sources, including glucose, fructose, galactose, glycerol, N-acetylglucosamine, and sucrose. The carbon source adaptability suggests the industrial potential of the strain, considering that the well-studied *P. putida* cannot utilize substrates such as sucrose to produce PHA without metabolic engineering. Finally, we optimized carbon and nitrogen sources, cultivation time, temperature, and inoculum size, and evaluated fructose syrup as a cost-effective feedstock for PHA production. In conclusion, *Pseudomonas* sp. B14-6 could be used for commercial PHA production with an economical starting material, resulting in a unique polymer that contains a very high proportion of mcl-PHA.

## Figures and Tables

**Table 1:** Monomeric units (mol %) of produced PHA.

Substrate	3HB	HHx	3HO	3HD	3HdDe	3HdD	3HtD
Glucose	5.89 ± 0.07	8.77 ± 0.16	29.09 ± 0.14	37.27 ± 0.03	8.56 ± 0.15	9.82 ± 0.16	0.60 ± 0.03
Glycerol	5.99 ± 0.75	8.51 ± 0.28	39.80 ± 1.06	33.55 ± 0.19	5.58 ± 0.05	6.46 ± 0.33	0.12 ± 0.02
Fructose	5.04 ± 0.15	7.75 ± 0.10	25.60 ± 0.05	40.03 ± 0.31	9.88 ± 0.03	11.01 ± 0.20	0.68 ± 0.02
Fructose syrup	0.83 ± 0.20	9.30 ± 1.05	38.43 ± 0.49	30.36 ± 1.17	13.33 ± 0.32	0.83 ± 0.20	ND

**Table 2:** Comparison of PHA production in psychrophilic *Pseudomonas* spp.

Strain	Substrate	Polymer Type	Content (%)	Volumetric Productivity (mg/L/h)	Reference
*Pseudomonas* sp. UMAB-40	Glucose	mcl-PHA	23	2.6	[41]
	Glycerol	mcl-PHA	11	1.6	[41]
	Sodium octanoate	mcl-PHA	48	9.4	[41]
*Pseudomonas* sp. PAMC 28620	Glycerol	mcl-PHA	52	24	[30]
*Pseudomonas mandelii CBS-1*	Fructose	scl-PHA	76	464	[42]
*Pseudomonas fluorescens BM07*	Fructose	mcl-PHA	25	23	[43]
*Pseudomonas* sp. B14-6	Fructose	scl-*co*-mcl PHA	35	4.6	This study
	Fructose syrup	scl-*co*-mcl PHA	24	6.4	This study

**Table 3:** Molecular weights and thermal properties of poly(3HB-*co*-3HA) samples.

	PHA Composition (mol %)		Molecular Weight			Thermal Properties			Reference
Sample	3HB (C_4_)	3HA (C_6_-C_12_)	M_n_ (×10^4^)	M_w_ (×10^4^)	PD	T_g_ (°C)	T_m_ (°C)	ΔH_m_ (J/g)	
P(3HA)_B14-6_	5	95	3.6	9.1	2.5	15.3	113	41.2	This study
P(3HA)_MPC6_	89.5	10.5	118	490	4.1	2.3	164	ND	[37]
P(3HA)_1_	44	56	4.0	139	4.4	−43	ND	0	[38]
P(3HA)_2_	88	12	34.9	48.9	1.4	−13	106	38	[47]
P(3HB)	100	0	65.0	117	1.8	4	178	91	[48]
LDPE						−30	130	220	[49]

## Data Availability

Data are contained within the article.
